# Exploring Parental Knowledge, Attitudes, and Factors Influencing Decision-Making in Stem Cell Banking: Rising the Future of Medical Treatment

**DOI:** 10.7759/cureus.58384

**Published:** 2024-04-16

**Authors:** Amani A. Alrehaili

**Affiliations:** 1 Department of Clinical Laboratory Sciences, College of Applied Medical Sciences, Taif University, Taif, SAU

**Keywords:** decision-making, factors, attitudes, knowledge, parents, stem cell banking

## Abstract

Background and objectives: Stem cell banking (SCB) is a promising area of modern medicine with the potential to yield innovative treatments and cures. To effectively educate parents and implement laws and regulations that address parental concerns and encourage informed decision-making, it is imperative to emphasize parental viewpoints and their consequences for future healthcare. The study aims to establish the Saudi Arabian population's level of understanding regarding SCB and to comprehend the elements influencing parental knowledge, attitudes, and SCB decision-making processes.

Methodology: A cross-sectional study was conducted among the population in the Makkah region of Saudi Arabia. Demographic data, knowledge levels, attitudes, and decision-making variables were gathered from 380 respondents.

Results: The study reveals a lack in their comprehension of the objectives and possible uses of SCB, together with the main sources of information on those banks and conveniently available banking choices. It showed varied results regarding attitudes about considering an SCB for their children. In addition, the majority of respondents had not made a consent decision about SCB for their children. It also illuminates the factors that could influence participants’ decisions about SCB for their children and shows that a lack of information and understanding is the main obstacle faced by parents regarding SCB. It highlights that participants were generally in favor of learning more about SCB for their children.

Conclusions: This study broadens our understanding of parental decision-making toward SCB and clarifies the elements influencing parents' opinions and worries and offers significant ramifications for lawmakers, medical professionals, and SCB. These implications can be utilized to enhance communication strategies, create instructional programs, and ease the fears of concerned parents.

## Introduction

The body has stem cells in a variety of organs and tissues, including the heart, brain, and muscles. Furthermore, stem cells can be produced in the lab (i.e., embryonic stem cells (ESCs) and induced pluripotent stem cells (iPSCs) or separated from the waste products of birth (cord blood (CB) and cord tissues) [1]. Throughout the body, stem cells are a population of undifferentiated cells with the capacity for perpetual self-renewal and the production of functional offspring with highly specialized cell functions. Depending on their location or tissue compartment, stem cells have different proliferative characteristics and roles. The capacity to self-renew and differentiate into all mature blood lineages is a defining characteristic of hematopoietic stem cells (HSCs) [2,3]. The continual developmental process known as hematopoiesis produces the various blood lineages by allowing HSCs to choose their own destiny as cells [4]. Another type of multifunctional stem cell that may be extracted and grown from the umbilical cord is called the umbilical cord mesenchymal stem cell (UC-MSC). They are poorly immunogenic, have multidirectional differentiation potential, and are strongly self-renewing. Numerous outcomes have been obtained from their use in the fields of tissue engineering and gene therapy, and their ability to prevent tumor cell multiplication and migration to the cancerous nest has been validated by recent investigations. The potential benefits of using UC-MSCs in the treatment of hematologic illnesses are evident in their capacity to suppress the immune system and promote the hematopoietic milieu. This may lead to improved engraftment following HSC transplantation [5].

Pluripotency is the extraordinary ability of ESCs to differentiate into any specialized cell type of the organism from which they originated. ESCs can, therefore, differentiate into any type of adult cell. To maintain pluripotency, ESCs must proliferate while suppressing differentiation, a process known as self-renewal [6]. Although adult stem cells are the ethically better option, there are not many sources of human adult stem cells, and the patient may experience difficult and unpleasant isolation. In contrast to human ESCs (hESCs), adult stem cells are lineage-restricted and have a limited ability to self-renew, which makes their proliferation in vitro extremely challenging [6]. With less debate, adult somatic cell-derived iPSCs are being researched more and more as a patient-specific substitute for hESCs. Crucially, there are many similarities between iPSCs and ESCs, which gives renewed hope for the use of pluripotent stem cells in regenerative therapies with less moral ambiguity and possibly better patient specificity [7,8].

The therapeutic and research potential of hESCs and human iPSCs can be attributed to their pluripotency, or the ability to differentiate into any of the cell types that make up the human body. In the context of regenerative medicine, ESC/iPSC proliferation and directed differentiation into homogenous cells ex vivo, followed by transplantation, are of great interest. In this way, ESC/iPSC-derived cell types would be used in transplants to replace or repair cells that have been lost or harmed as a result of illness or trauma in order to implement regenerative medicine techniques [6]. Generally, ESCs and iPSCs have their unique characteristics and applications. Their potential to generate replacement cells, model diseases, and develop personalized therapies offers hope for treating a wide range of medical conditions and improving patient outcomes [6].

Adipose tissue (AT), umbilical cord blood (UCB) and tissue, and bone marrow (BM) are all useful sources of stem cells; they include stem cells that are generally accessible to most people, easily procured, and reasonably priced. The ability to cryogenically store stem cells in their most effective form for future use is provided by stem cell banking (SCB) [1]. After giving birth, the blood in the placenta and umbilical cord has several benefits and is similar to BM in terms of its suitability for HSC transplantation [9]. CB also includes a mixture of pluripotent stem cells that can give rise to cells originating from the endodermal, mesodermal, and ectodermal lineages, according to research conducted by multiple independent laboratories [10,11].

A promising field of modern medicine in terms of producing ground-breaking cures and treatments is SCB [1,9]. In order to assist any patient in need of a stem cell transplant, more than 700000 UCB units are currently kept in public CB banks across the globe, two of which are in Saudi Arabia, where no private banks are permitted due to legal restrictions [12]. For families with a history of hematological disorders, both of the Saudi public banks provide specialized banking services. Families that would prefer to store their UCB in private banks and move it to foreign storage facilities can also take advantage of a number of private companies' collection services [12]. UCB is preserved upon request; it is not done on a regular basis. Therefore, people should be aware of this once-in-a-lifetime chance to gather and preserve UCB for the range of therapeutic modalities it may provide, whether for themselves, their families, or society at large [13]. However, the successful application of stem cells in medical treatments depends on the availability of viable and well-preserved samples. In this case, parents’ decision-making and level of understanding play a critical role in determining whether stem cells are banked and, if so, how they may impact future medical interventions.

Numerous studies of people’s knowledge, comprehension, preferences, and attitudes regarding CB banking have been carried out in different countries. The majority of those findings indicate a lack of public awareness, with mothers and even medical professionals and other healthcare providers assumed to be knowledgeable about it [14-23]. Therefore, the primary purpose of the present study is to determine the level of knowledge of SCB among the Saudi Arabian population. Followed by understanding the elements influencing parental knowledge, attitudes, and SCB decision-making processes, which is essential for developing effective strategies for teaching parents and implementing laws and regulations that address parental concerns and promote informed decision-making [24]. Stakeholders in the healthcare system can address parents’ knowledge gaps, fears, and decision-making considerations by working to enhance SCB’s potential advantages and use. Prior studies have examined several facets of SCB, including its advantages for science and medicine, but little attention has been paid to the specific role that parental comprehension and decision-making play [1]. The present study seeks to bridge this gap by focusing on parental perspectives and their implications for future healthcare. By conducting a thorough analysis of parental awareness, decision-making factors, concerns, and information sources, the aim is to provide actionable information to legislators, healthcare professionals, and SCBs. Future healthcare will be significantly impacted by the study’s findings. Interested parties can develop educational initiatives that address concerns related to SCB, promote informed decision-making, and increase public awareness by analyzing parental knowledge and decision-making styles. The study seeks to advance healthcare practices in SCB through the expanded use of stem cells in medicinal interventions that lead to better patient outcomes.

## Materials and methods

Study design and settings

A cross-sectional study was conducted among the population in the Makkah region of Saudi Arabia. Saudi and non-Saudi parents in the Makkah region were included in the study and those from other regions were excluded. The larger study involved 602 participants, but a total of 380 responses were included in the present study.

Sample size calculation and study sample size

Cochrane’s sample size equation (n=z2pq/e2) was used to determine an appropriate sample size, where n denotes the smallest sample size, z denotes the desired confidence interval (95%), p stands for proportion, q=1p, and e stands for the necessary degree of precision (5%). The population proportion used to determine the sample size for this investigation was 55% (p). The estimated sample size was 362 based on that formula. We collected around 36.88% additional data to compensate for the loss of data due to invalid forms or missing responses. A total of 602 individuals accessed the survey link for the questionnaire, of whom 380 were included in the final sample.

Ethical considerations

This study was approved by Taif University’s Ethics Committee under application number HAO-02-T-105. All participants were asked if they agreed to participate in this study prior to beginning, and all data and other information were kept confidential.

Data collection instrument

A structured questionnaire was created to survey the sample of parents; it consisted of multiple-choice questions and demographic data. Knowledge levels, decision-making variables, concerns, and demographic data were gathered through the electronic distribution of the questionnaire. The survey consisted of five sections: section 1 related to demographic information (age, gender, having children, educational levels, and residential area in Saudi Arabia); section 2 asked four questions related to knowledge and awareness of SCB (Have you heard about stem cell banking? Do you understand the purpose and potential applications of a stem cell bank? What are the most commonly used stem cell sources for a stem cell bank? Which of the following stem banking centers options have you heard of?). Section 3 had seven questions related to decision-making attitudes (i.e., considerations, factors, concerns, and education about SCB): Have you looked into a stem cell bank for your child? Have you made a consent decision about stem cell banking for your child? Are you currently considering a stem cell bank for your children? What factors are influencing or will influence your decision about stem cell banking for your children? How did you get information or education about stem cell banking for your children? How do you rate your level of knowledge about stem cell banking for your children? Would you like to receive more information about stem cell banking for your children?

The study’s survey questions were designed to capture the most crucial information required to assess trends in knowledge, opinions, and attitudes. With the assistance of knowledgeable researchers and experts in the field, the study’s logical and content validity in relation to its aims was confirmed. After that, the survey was translated into Arabic so that the researchers could better grasp its contents. With the assistance of bilingual Arabic-English speakers, the Arabic translation was compared to the English version for logical and construction accuracy. Additionally, a pilot study with a small sample of the population was conducted to see whether any points were missing or if respondents had any problems understanding the questionnaire items. Data were gathered via an electronic survey form that displayed the questions detailed above. Among the 602 participants, 386 were deemed eligible based on their parental status and residency in the Makkah region of Saudi Arabia. Responses from participants whose demographic attributes did not align with the study target were excluded.

Data analysis

The collected data were analyzed using Prism 10 Version 10.1.1. Frequency and percentages were used to report categorical data, whereas ratios and demographic characteristics were compared using the chi-square test. A statistically significant value was defined as p<0.05.

## Results

Assessment of demographic characteristics

Among the 380 respondents who met the eligibility criteria, 74.55% (n=287) were between the ages of 40 and 59, followed by 16.36% (n=63) in the 30-39 age range and 9.09% between 20 and 29. Nearly three-quarters (74.55%, n=287) were female; 25.45% (n=98) were male. The responses also showed that 43.64% (n=168) of the respondents had postgraduate education, 41.82% (n=161) had undergraduate degrees, and just 14.55% (n=56) of the respondents had completed high school (see Table 1).

**Table 1:** Distribution of demographic data of the participants

Demographics	Frequency	%
Age		
40-59	287	74.55
30-39	63	16.36
20-29	35	9.09
Total	385	100
Gender		
Female	287	74.55
Male	98	25.45
Total	385	100
Educational level		
Postgraduate	168	43.64
Undergraduate	161	41.82
High school	56	14.55
Total	385	100

Assessment of stem cell banking knowledge

Table 2 displays the frequency and proportion of participants’ responses to the knowledge domain items. The majority of respondents (72.73%, n=280) had heard about SCBs, while 56.36% (n=217) of those aged 40 to 59 and 54.55% (n=210) of females had done so. However, there were no significant differences for age and gender (p=0.073 and p=0.11, respectively). An analysis of education levels showed significant differences (p<0.001) for those with postgraduate education (38.18%, n=147) and those with undergraduate (27.27%, n=105) and (7.27%, n=28) high school education, as shown in Table 2.

**Table 2:** Distribution of demographic data of the participants’ knowledge - Q1

Q1. Have you heard about stem cell banking?						
Demographics	Yes	No	Total	Chi^2^	df	p-value
Age						
40-59	217 (56.36%)	70 (18.18%)	287 (74.55%)	5.23	2	0.073
30-39	42 (10.91%)	21 (5.45%)	63 (16.36%)			
20-29	21 (5.45%)	14 (3.64%)	35 (9.09%)			
Total	280 (72.73%)	105 (27.27%)	385 (100%)			
Gender						
Female	210 (54.55%)	77 (20%)	287 (74.55%)	0.739	1	0.11
Male	70 (18.18%)	28 (7.27%)	98 (25.45%)			
Total	280 (72.73%)	105 (27.27%)	385 (100%)			
Educational level						
Postgraduate	147 (38.18%)	21 (5.45%)	168 (43.64%)	37.65	2	<0.001
Undergraduate	105 (27.27%)	56 (14.55%)	161 (41.82%)			
High school	28 (7.27%)	28 (7.27%)	56 (14.55%)			
Total	280 (72.73%)	105 (27.27%)	385 (100%)			

The following knowledge item assessed the participants' understanding of the purpose and potential applications of SCB. Of respondents aged 40-59 (74.55%, n=287), approximately 30.91% (n=119) moderately understood SCB but needed more information; 23.64% (n=91) of them understood it and 20% (n=77) did not know. Additionally, the analysis of gender for the same cohort showed that 105 (27.27%) were female and 56 were male (14.55%); both showed a moderate understanding but required more information. The comparison is statistically significant (p=0.002; see Table 3). Focusing on the participants’ educational levels, among 41.82% (n=161) of participants, around 23.64% (n=91) aged 30-39 were moderately informed about the purpose and application of SCBs but needed more information; 5.45% (n=21) and 12.73% (n=49) knew well or did not know, respectively. These results are statistically significant when compared to other groups (p<0.001; see Table 3).

**Table 3:** Distribution of demographic data of the participants’ knowledge - Q2

Q2. Do you understand the purpose and potential applications of a stem cell bank?								
Demographics		To some extent, but I would like more information	Yes	No	Total	Chi^2^	df	p-value
Age								
40-59		119 (30.91%)	91 (23.64%)	77 (20%)	287 (74.55%)	18.27	4	0.001
30-39		35 (9.09%)	7 (1.82%)	21 (5.45%)	63 (16.36%)			
20-29		7 (1.82%)	14 (3.64%)	14 (3.64%)	35 (9.09%)			
Total		161 (41.82%)	112 (29.09%)	112 (29.09%)	385 (100%)			
Gender								
Female		105 (27.27%)	91 (23.64%)	91 (23.64%)	287 (74.55%)	12.69	2	0.002
Male		56 (14.55%)	21 (5.45%)	21 (5.45%)	98 (25.45%)			
Total		161 (41.82%)	112 (29.09%)	112 (29.09%)	385 (100%)			
Educational level								
Postgraduate		56 (14.55%)	84 (21.82%)	28 (7.27%)	168 (43.64%)	90.31	4	<0.001
Undergraduate		91 (23.64%)	21 (5.45%)	49 (12.73%)	161 (41.82%)			
High school		14 (3.64%)	7 (1.82%)	35 (9.09%)	56 (14.55%)			
Total		161 (41.82%)	112 (29.09%)	112 (29.09%)	385 (100%)			

Assessing the knowledge of participants regarding the most commonly used stem cell sources for the SCB showed that 98 (25.45%) of the 40-59 age group knew about CB, followed by 91 (23.64%) account for BM, while 49 (12.73%) in the 30-39 age group year knew about CB and seven (1.82%) knew about BM; only 14 (3.64%) of the 20-29 age group knew about CB, followed by seven (1.82%) for BM. For each age cohort, a few responded with other options (i.e., ESCs, dental pulp (DP), iPSCs, ATs, and other unspecified sources). These results are statistically significant when compared to other cohort groups (p<0.001; see Table 4). For gender analysis, 126 (32.73%) females knew about CB, followed by 77 (20%) for BM. When compared to males, this result is statistically significant (p<0.001; see Table 4). For educational levels, around 84 (21.82%) of postgraduate, 63 (16.36%) of undergraduate, and 14 (3.64%) of high school knew about CB; meanwhile 49 (12.73%), 42 (10.91%), and 14 (3.64%) of the respective educational groups knew about BM. These results are statistically significant (p<0.001; see Table 4). 

**Table 4:** Distribution of demographic data of the participants’ knowledge - Q3 CB, cord blood; ESCs, embryonic stem cells; DP, dental pulp; iPSCs, induced pluripotent stem cells; ATs, adipose tissues

Q3. What are the most commonly used stem cell sources for the stem cell bank?											
Demographics	CB	ESCs	BM	DP	Others	iPSCs	ATs	Total	Chi^2^	df	p-value
Age											
40-59	98 (25.45%)	63 (16.36%)	91 (23.64%)	0 (0%)	14 (3.64%)	14 (3.64%)	7 (1.82%)	287 (74.55%)	141.89	12	<0.001
30-39	49 (12.73%)	0 (0%)	7 (1.82%)	0 (0%)	7 (1.82%)	0 (0%)	0 (0%)	63 (16.36%)			
20-29	14 (3.64%)	0 (0%)	7 (1.82%)	7 (1.82%)	7 (1.82%)	0 (0%)	0 (0%)	35 (9.09%)			
Total	161 (41.82%)	63 (16.36%)	105 (27.27%)	7 (1.82%)	28 (7.27%)	14 (3.64%)	7 (1.82%)	385 (100%)			
Gender											
Female	126 (32.73%)	42 (10.91%)	77 (20%)	7 (1.82%)	28 (7.27%)	7 (1.82%)	0 (0%)	287 (74.55%)	40.25	6	<0.001
Male	35 (9.09%)	21 (5.45%)	28 (7.27%)	0 (0%)	0 (0%)	7 (1.82%)	7 (1.82%)	98 (25.45%)			
Total	161 (41.82%)	63 (16.36%)	105 (27.27%)	7 (1.82%)	28 (7.27%)	14 (3.64%)	7 (1.82%)	385 (100%)			
Educational level											
Postgraduate	84 (21.82%)	14 (3.64%)	49 (12.73%)	0 (0%)	14 (3.64%)	0 (0%)	7 (1.82%)	168 (43.64%)	103.58	12	<0.001
Undergraduate	63 (16.36%)	28 (7.27%)	42 (10.91%)	0 (0%)	14 (3.64%)	14 (3.64%)	0 (0%)	161 (41.82%)			
High school	14 (3.64%)	21 (5.45%)	14 (3.64%)	7 (1.82%)	0 (0%)	0 (0%)	0 (0%)	56 (14.55%)			
Total	161 (41.82%)	63 (16.36%)	105 (27.27%)	7 (1.82%)	28 (7.27%)	14 (3.64%)	7 (1.82%)	385 (100%)			

The participant’s knowledge regarding the available options for SCB centers showed that 210 (54.55%) of respondents were not aware of them. Focusing on the 40-59 age group, 168 (43.64%) were not aware, followed by 63 (16.36%) for public SCB and 35 (9.09%) for private SCB. Similar trends were observed in the 30-39 group (28 (7.27%) were not aware, 63 (16.36%) knew of public SCB, and 35 (9.09%) knew about private SCB). The comparison between the age cohort groups was statistically significant (p<0.001; see Table 5). For gender analysis, 168 (43.64%) of females were not aware of the available options for SCBs, followed by 63 (16.36%) regarding public SCB, and 49 (12.73%) regarding private SCB; these results are statistically significant (p<0.001) when compared to the results of the male group, as shown in Table 5.

**Table 5:** Distribution of demographic data of the participants’ knowledge - Q4 SCB, stem cell banking; TS-SCB, tissue-specific stem cell banking

Q4. Which of the following stem banking center options have you heard of?								
Demographics	Not aware	Public SCB	Private SCB	TS-SCB	Total	Chi^2^	df	p-value
Age								
40-59	168 (43.64%)	63 (16.36%)	35 (9.09%)	21 (5.45%)	287 (74.55%)	30.56	6	<0.001
30-39	28 (7.27%)	21 (5.45%)	14 (3.64%)	0 (0%)	63 (16.36%)			
20-29	14 (3.64%)	14 (3.64%)	0 (0%)	7 (1.82%)	35 (9.09%)			
Total	210 (54.55%)	98 (25.45%)	49 (12.73%)	28 (7.27%)	385 (100%)			
Gender								
Female	168 (43.64%)	63 (16.36%)	49 (12.73%)	7 (1.82%)	287 (74.55%)	61.65	3	<0.001
Male	42 (10.91%)	35 (9.09%)	0 (0%)	21 (5.45%)	98 (25.45%)			
Total	210 (54.55%)	98 (25.45%)	49 (12.73%)	28 (7.27%)	385 (100%)			
Educational level								
Postgraduate	63 (16.36%)	56 (14.55%)	42 (10.91%)	7 (1.82%)	168 (43.64%)	93.7	6	<0.001
Undergraduate	98 (25.45%)	42 (10.91%)	0 (0%)	21 (5.45%)	161 (41.82%)			
High school	49 (12.73%)	0 (0%)	7 (1.82%)	0 (0%)	56 (14.55%)			
Total	210 (54.55%)	98 (25.45%)	49 (12.73%)	28 (7.27%)	385 (100%)			

In terms of participants’ educational levels, the results showed that 210 (54.55%) were not aware of the centers. Roughly a quarter (n=98, 25.45%) at the undergraduate level were not aware of the centers, followed by 42 (10.91%) aware of public SCB; 21 (5.45%) were aware of tissue-specific stem cells, and none was aware of private SCB (0%). When compared to the postgraduate level of participants, around 63 (16.36%) were not aware of the centers, followed by 56 (14.55%) for public SCBs and 42 (10.91%) for private SCBs. These results are statistically significant (p<0.001), as presented in Table 5.

Assessment of stem cell banking attitudes

Different assessments related to participants’ attitudes were also part of the present study. The majority of respondents were not looking at SCB for their children (90.91%, n=350; see Table 6A). In addition, 96.36% (n = 371) of respondents had not made a consent decision about SCB for their children; only 3.64% (n = 14) of respondents had done so (Table 6B). The results varied regarding attitudes about considering an SCB for their children; about 40%, (n = 154) of respondents were not sure, 30.09% (n = 119) had not considered SCB, and only 29.09% (n = 112) were considering SCB (Table 6C).

**Table 6:** Assessments of study participants’ attitudes

Question	Option	Frequency	%
A. Have you looked into a stem cell bank for your child?	No	350	90.91
	Yes	35	9.09
	Total	385	100
B. Have you made a consent decision about stem cell banking for your child?	No	371	96.36
	Yes	14	3.64
	Total	385	100
C. Are you currently considering a stem cell bank for your child or children?	Not Sure	154	40
	No	119	30.91%
	Yes	112	29.09%
	Total	380	100%
D. What factors will influence your decision about stem cell banking for your child or children?	Lack of information or understanding	140	36.36
	Potential future medical benefits	105	27.27
	Cost of bank and storage	77	20
	The ethical considerations	28	7.27
	Information and testimonials from other parents	28	7.27
	Recommendations from health professionals	7	1.82
	Total	385	100
E. How have you obtained information or education about stem cell banking for your child or children?	Health professionals	105	27.27
	I received no information or education	98	25.45
	Internet or social media	91	23.64
	Family or friends	49	12.73
	Educational institutions	42	10.91
	Total	385	100
F. How do you rate your level of knowledge about stem cell banking for your child or children?	Not knowledgeable at all	154	40
	Fairly knowledgeable	112	29.09
	Moderately knowledgeable	98	25.45
	Very knowledgeable	21	5.45
	Total	380	100%
G. Would you like to receive more information about stem cell banking for your child/children?	Yes	336	87.27
	No	49	12.73
	Total	385	100

The factors that could influence participants’ decision about SCBs for their children were analyzed and showed that 36.36% (n=140) of participants lacked enough information or understanding, followed by a positive attitude among respondents related to potential future medical benefits; this percentage was 27.27% (n=105). The next factor covered the costs of cell banks and storage, which accounted for 20% (n=77) of participants; the factors of ethical considerations and information and testimonials from other parents accounted for 7.27% (n=28) in each case. The least important factor was recommendations from health professionals (1.82%, n=7; Table 6D).

Subsequently, the participants’ attitudes regarding the information or education about SCB for their children were assessed and showed a range of results. Health professionals accounted for 27.27% (n=105), followed by 25.45% (n=98) who received no information or education, 23.64% (n=91) who received information from social media or elsewhere on the internet, 12.73% (n=49) from family or friends, and 10.91% (n=42) from educational institutions (Table 6E).

The levels of participant knowledge about SCBs for their children were also examined and showed that a majority were not knowledgeable at all (40%, n=154), while 29.09% (n=112) were fairly knowledgeable, 25.45% (n=98) were moderately knowledgeable, and only 5.45% (n=21) were very knowledgeable (Table 6F). Regardless of their current levels of awareness, participants were overwhelmingly positive about receiving more information about SCBs for their children, with 87.27% (n=336) in favor and only 12.73% (n=49) opposed (Table 6G).

## Discussion

Given the potential promise of stem cell-based medicine, the availability of viable, well-preserved samples is essential for the effective use of stem cells in medical treatments. In this instance, whether stem cells are banked and, if so, how they might affect upcoming medical procedures depends greatly on the parents’ decision-making and comprehension levels. Thus, the goal of the present study was to ascertain the level of knowledge of the population of one Saudi region about SCB in Saudi Arabia and to comprehend their understanding and the factors affecting parents’ attitudes toward decision-making about SCB. That will enable the creation of successful parent education programs and the implementation of laws and regulations that respond to parental concerns and encourage wise decision-making.

Finding out the levels of parental knowledge and comprehension regarding SCB was the first stage of this study. Of 380 respondents, 72.73% had heard of SCB; around 56.36% were in the 40-59 age range, 54.55% were female, and 147 (38.18%) had postgraduate education. Having heard about SCB could be explained by the high percentage of educated participants in the present study. These results are in line with Jordens et al., 2014, who showed that Australia had a higher level of awareness, with 70% (n=1873) of the sample population recognizing CB banking [21]. In the present study, the understanding of the purpose and potential applications of SCB showed that within one age group (40-59), roughly 30.91% (n=119) understood the purposes and applications of SCB but needed further knowledge; these results were statistically significant when compared to other groups. In addition, the current study has shown significant differences in participant education; a higher percentage of them held undergraduate degrees (23.64%, n=91) compared to other educational levels. These findings suggest that even though the majority of parents had heard about SCB, they did not fully comprehend its significance or its possible uses in the future. These results may correlate with the study by Katz et al., 2011, who investigated the knowledge of pregnant women about CB SCB options in France, Germany, Italy, Spain, and the United Kingdom. Their study showed that 79% (n=1620) of expectant mothers reported knowing very little about CB banking [15]. Moreover, these findings accord with reports that people in a number of nations lack sufficient understanding regarding CB banking [22,23]. Similar findings were reported in a recent survey conducted in India, where only 26% of people were familiar with CB banking [19]. The results of the present study also accord with Grano et al., 2020, who demonstrated that while most women in their study were aware that CB donation was an option, most did not have enough information in general, know what protocols to follow when donating, or understand the potential applications for UCB in the clinic and in the lab. The vast majority of these women wished they had known more about UCB donation and storage [25].

The present study also evaluated the level of parental knowledge of the most commonly used sources for SCB. It is well known that replacement BM components can be obtained from CB, which is a rich source of hematopoietic cells. Regenerative medicine is increasingly using stem cells to treat a variety of systemic ailments and blood abnormalities. In order to facilitate allogeneic or autologous transplantation, collected blood is currently kept in public or commercial banks [26]. In October 1988, the first hematopoietic transplant using CB as the source of hematopoietic cells was carried out; CB banking has now advanced to the point where more than four million units are kept in commercial banks globally, and about 800000 units are kept in public banks [27]. However, the present study showed that less than half of participating parents (41.82%, n=161) knew that the correct source for SCB was CB; that was true for only 98 (25.45%) of the 40-59 age group, 49 (12.73%) of the 30-39 year cohort, and only 14 (3.64%) of those aged 20 to 29, although 84 (21.82%) had postgraduate education. These results accord with a study by Jawdat et al., 2018, that investigated public awareness in Saudi Arabia on CB banking and showed that only half of the subjects (52%, n=601) knew that it was a source of stem cells [12].

CB banking is a well-established and regulated process that has been used in thousands of regenerative therapies and over 30000 stem cell transplants over the past 20 years [1]. An evaluation of parents’ understanding of accessible SCB options showed that more than half of the participants (54.55%, n=210) were not aware of SCB centers, many of whom were females between 40 and 59 (43.64%, n=168). In addition, 98 (25.45%) had undergraduate education and 16.36 (n=63) had postgraduate education. Only 25.45% (n=98) of the total respondents knew about public CB banking. These results agree with a previous study by Fernandez et al., which showed that the majority of the women (70% and 74%) expressed having an inadequate or extremely limited understanding of CB banking centers, respectively [22]. Thus, although the present study focused on parents who were highly educated, the results showed they had poor knowledge of the purposes and potential applications of SCB, the main sources of stem cells, and accessible banking options, all of which demonstrate the need for educational initiatives and awareness programs.

In order to explore the possible future use of SCB among the parents included in the study, their attitudes were also assessed, beginning with whether they looked into an SCB for their children. The results revealed that the vast majority had not looked at SCB for their children (90.91%, n=350). Subsequently, their attitudes toward making a consent decision about SCB for their children are important to facilitate focusing on correlating their knowledge with possible obstacles to their making such a decision. These results also weighed heavily in one direction: 96.36% (n=371) had not made a consent decision regarding their children and SCB; only 3.64% (n=14) had done so. Overall, these findings may be explained by their poor knowledge of SCB, especially their lack of understanding of their purposes and potential applications, the main sources for SCB, and accessible banking options. Thus, the next attitude assessment focused on whether they were currently considering SCB for their children as a result of being part of the present study. The results were varied: about 40% (n=154) of respondents were not sure, 30.91% (n = 119) were not considering SCB, and only 29.09% (n = 112) were considering SCB. Notably, the percentage of parents increased from those considering an SCB for their children (3.64%, n=14) to those who would not (29.09%, n = 112) and to those who were unsure (40%, n=154), meaning that there was a chance they would do so, highlighting the need for education and awareness on the whole idea of SCB.

Subsequently, the factors influencing parents' decisions on whether to bank their children’s stem cells (or not) were examined. The results showed significant differences: the reasons related to a lack of information or understanding, at 36.36% (n=140) of participants, were more important than other factors. This result could support the above analysis related to people’s need for more information regarding the purposes and potential applications of SCB, their poor knowledge of the most commonly used sources for SCB, and the available centers. The other factors were related to potential future medical benefits (27.27%, n=105), bank and storage costs (20%, n=77), ethical considerations, and information and testimonials from other parents, at 7.27% (n=28) each. The least important factor was recommendations from health professionals at 1.82% (n=7), which may be due to medical staff members’ lack of understanding, as was shown in a study by Walker et al., preventing them from confidently guiding discussions regarding CB banking [28]. Hence, as mentioned above, parental education programs are required, as is increasing health professionals’ knowledge and communication with the general population, in order to effectively educate their patients and encourage them to make an informed decision regarding their children’s CB. By offering educational sessions to medical personnel, patients and their families would have better awareness from a reliable, non-profit source.

This study also investigated the sources of information and guidance that parents consult when deciding whether to bank their children’s SCs. Their attitudes regarding the information and education about SCBs for their children were also assessed and showed varied results. Health professionals accounted for 27.27% (n=105), which may support increasing health professionals’ training and education about SCB. This accords with other studies showing that a majority of respondents acquired their information from medical experts [12,15]. By contrast, this result does not agree with a study showing that only 15% of respondents obtained their knowledge from health experts, with the majority receiving it from social media [29]. The other results are as follows: 25.45% (n=98) received no information or education, 23.64% (n=91) obtained it from the internet or social media, 12.73% (n=49) from family or friends, and only 10.91% (n=42) from educational institutions. Healthcare professionals, social and digital media, and families and friends, among other groups, can increase the knowledge and influence the opinions of parents. Identifying the most influential sources can guide the development of targeted educational activities and communication methods.

A direct attitude assessment related to participants’ knowledge about SCBs for their children was carried out and revealed meaningful differences. A large plurality of respondents were not knowledgeable at all (40%, n=154), while 29.09% (n=112) were fairly knowledgeable, 25.45%, (n=98) were moderately knowledgeable, and only 5.45% (n=21) were very knowledgeable. Since the majority of participants were female, it is recommended that physicians treating pregnant patients presume that they have limited knowledge regarding CB banking. Ensuring that every pregnant woman has the opportunity to make an informed decision regarding SCB should be the main objective of the decision-making process. There was a generally positive attitude among participants to receiving more information about SCB for their children. However, there was a generally positive attitude among participants about receiving more information about SCB for their children, with 87.27% (n=336) in favor and only 12.73% (n=49) opposed. There is thus a need for educational initiatives and public awareness efforts to advance attitudes and knowledge about stem cells among parents, especially regarding CB cells.

## Conclusions

The findings of this study increase our understanding of the role of parental decision-making in aiding the purposes of SCsB. It elaborates on the levels of parental understanding and the factors affecting their attitudes toward decision-making about SCsB. This may be the first study focused on parents in Saudi Arabia as to their decisions in facilitating SCsB. The study’s findings indicate that their understanding of the purposes and potential applications of SCsB, as well as the primary sources for those banks and easily accessible banking options, was lacking, indicating the need for increased awareness and education regarding SCsB. It also elucidates that a lack of information and understanding is the main obstacle faced by parents regarding SCsB. Significantly, they relied on health professionals for information and guidance and were generally in favor of learning more about SCsB for their children. Collectively, the study has important implications for legislators, healthcare providers, and SCsB because they can be used to improve communication strategies, develop educational initiatives, and allay parental worries. Understanding these issues and challenges is essential to effectively addressing them, providing parents with the necessary information, and motivating them. The study limitations could be the number of males in the study was lower than the number of females. Thus, expanding the present study among all regions of Saudi Arabia may be required to gather more data and support or refine its findings, thereby effectively enhancing SCB use in the future.

## References

[1] Harris DT (2014). Stem cell banking for regenerative and personalized medicine. Biomedicines.

[2] Bryder D, Rossi DJ, Weissman IL (2006). Hematopoietic stem cells: the paradigmatic tissue-specific stem cell. Am J Pathol.

[3] Shizuru JA, Negrin RS, Weissman IL (2005). Hematopoietic stem and progenitor cells: clinical and preclinical regeneration of the hematolymphoid system. Annu Rev Med.

[4] Ogawa M (1993). Differentiation and proliferation of hematopoietic stem cells. Blood.

[5] Shang Y, Guan H, Zhou F (2021). Biological characteristics of umbilical cord mesenchymal stem cells and its therapeutic potential for hematological disorders. Front Cell Dev Biol.

[6] Kingham E, Oreffo RO (2013). Embryonic and induced pluripotent stem cells: understanding, creating, and exploiting the nano-niche for regenerative medicine. ACS Nano.

[7] Condic ML, Rao M (2008). Regulatory issues for personalized pluripotent cells. Stem Cells.

[8] Amabile G, Meissner A (2009). Induced pluripotent stem cells: current progress and potential for regenerative medicine. Trends Mol Med.

[9] Butler MG, Menitove JE (2011). Umbilical cord blood banking: an update. J Assist Reprod Genet.

[10] McGuckin CP, Forraz N, Baradez MO (2005). Production of stem cells with embryonic characteristics from human umbilical cord blood. Cell Prolif.

[11] McGuckin CP, Forraz N, Allouard Q, Pettengell R (2004). Umbilical cord blood stem cells can expand hematopoietic and neuroglial progenitors in vitro. Experimental Cell Research.

[12] Jawdat D, AlTwijri S, AlSemari H, Saade M, Alaskar A (2018). Public awareness on cord blood banking in Saudi Arabia. Stem Cells Int.

[13] Knoppers BM, Isasi R (2010). Stem cell banking: between traceability and identifiability. Genome Med.

[14] Suen SS, Lao TT, Chan OK (2011). Maternal understanding of commercial cord blood storage for their offspring - a survey among pregnant women in Hong Kong. Acta Obstet Gynecol Scand.

[15] Katz G, Mills A, Garcia J (2011). Banking cord blood stem cells: attitude and knowledge of pregnant women in five European countries. Transfusion.

[16] Alhadlaq A, Al-Maflehi N, Alzahrani S, AlAssiri A (2019). Assessment of knowledge and attitude toward stem cells and their implications in dentistry among recent graduates of dental schools in Saudi Arabia. Saudi Dent J.

[17] Almaeen A, Wani FA, Thirunavukkarasu A (2021). Knowledge and attitudes towards stem cells and the significance of their medical application among healthcare sciences students of Jouf University. PeerJ.

[18] Al-Shammary AA, Hassan SU (2023). Inconsistencies in pregnant mothers’ attitudes and willingness to donate umbilical cord stem cells: a cross-sectional analysis from Saudi Arabia. J Clin Med.

[19] Pandey D, Kaur S, Kamath A (2016). Banking umbilical cord blood (UCB) stem cells: awareness, attitude and expectations of potential donors from one of the largest potential repository (India). PloS one.

[20] Karagiorgou LZ, Pantazopoulou MN, Mainas NC, Beloukas AI, Kriebardis AG (2014). Knowledge about umbilical cord blood banking among Greek citizens. Blood Transfus.

[21] Jordens CF, Kerridge IH, Stewart CL (2014). Knowledge, beliefs, and decisions of pregnant Australian women concerning donation and storage of umbilical cord blood: a population-based survey. Birth.

[22] Fernandez CV, Gordon K, Van den Hof M, Taweel S, Baylis F (2003). Knowledge and attitudes of pregnant women with regard to collection, testing and banking of cord blood stem cells. Can Med Assoc.

[23] Lu H, Chen Y, Lan Q (2015). Factors that influence a mother’s willingness to preserve umbilical cord blood: a survey of 5120 Chinese mothers. PLoS One.

[24] Peberdy L, Young J, Massey DL, Kearney L (2018). Parents' knowledge, awareness and attitudes of cord blood donation and banking options: an integrative review. BMC Pregnancy Childbirth.

[25] Grano C, Scafa V, Zucaro E, Abad R, Lombardo C, Violani C (2020). Knowledge and sources of information on umbilical cord blood donation in pregnant women. Cell Tissue Bank.

[26] Devi S, Bongale AM, Tefera MA, Dixit P, Bhanap P (2023). Fresh umbilical cord blood—a source of multipotent stem cells, collection, banking, cryopreservation, and ethical concerns. Life.

[27] Mayani H, Wagner JE, Broxmeyer HE (2020). Cord blood research, banking, and transplantation: achievements, challenges, and perspectives. Bone Marrow Transplant.

[28] Walker T, Steckler D, Spellman S, Haven D, Welte K, Boo M (2012). Awareness and acceptance of public cord blood banking among practicing obstetricians in the United States. Transfusion.

[29] Velikonja NK, Erjavec K, Knežević M (2021). Knowledge, Awareness, and Attitudes toward Umbilical Cord Blood Biobanking. Open Access Maced J Med Sci.

